# Marginal cord insertion among singleton births at the University of Gondar Comprehensive Specialized Hospital, Northwest Ethiopia

**DOI:** 10.1186/s12884-021-03703-x

**Published:** 2021-03-17

**Authors:** Hailu Aragie, Mohammed Oumer

**Affiliations:** 1 Department of Human Anatomy, School of Medicine, College of Medicine and Health Science, University of Gondar, Gondar, Ethiopia; 2 Department of Epidemiology, Institute of Public Health, College of Medicine and Health Sciences, University of Gondar, Gondar, Ethiopia

**Keywords:** Marginal cord insertion, Adverse birth outcomes, Singleton births, Gondar, Ethiopia

## Abstract

**Background:**

Umbilical cord may insert abnormally i.e. marginal insertion to a placenta which can cause different birth and perinatal complications. Despite the increased effort taken by different responsible bodies, the prevalence of birth and perinatal complications are still high, possibly due to anomalous cord insertion. So far, anomalous cord insertion lacks proper attention in different medical settings. Hence, the present study aims to assess the magnitude, risk factors, and adverse birth outcomes of marginal cord insertion among singleton births.

**Methods:**

An institution-based cross-sectional study design was conducted. A systematic random sampling technique was used to select study participants. Data were collected by using a structured questionnaire and it was entered into epi-data version 3.1 then exported to SPSS version 20 for data cleansing and analysis. Bi-variable and multivariable logistic regressions were employed to identify risk factors and adverse outcomes associated with marginal cord insertions. Crude and adjusted odds ratio (*P*-value < 0.05) with a 95% confidence interval were calculated.

**Result:**

The magnitude of marginal cord insertion was 6.4% (95% CI = 4.4–8.8%) in singleton pregnancies. Independent risk factors for marginal cord insertion were advanced maternal age (AOR = 2.24, 95% CI: 1.35–11.08), primiparity (AOR = 1.98, 95% CI: 1.37–8.69), maternal chronic hypertension (AOR = 3.07, 95% CI: 1.66–9.76), previous cesarean delivery (AOR = 2.51, 95% CI: 1.43–10.21), and use of intrauterine contraceptive device before pregnancy (AOR = 2.22, 95% CI: 1.36–12.30). Pregnancies complicated by marginal cord insertion are at higher risk to develop low birth weight (AOR = 2.89, 95% CI: 1.23–6.80), preterm birth (AOR = 4.00, 95% CI: 1.44–11.14), and emergency cesarean delivery (AOR = 3.68, 95% CI: 1.03–13.81).

**Conclusion and recommendation:**

Marginal cord insertion is a mistreated potential risk for low birth weight, preterm birth, and emergency cesarean delivery. Routine screening of marginal cord insertion should be considered in pregnancies with advanced age, nulliparity, hypertensive disorder, history of cesarean section, and intrauterine contraceptive device usage before pregnancy.

## Introduction

The umbilical cord is a cord-like structure, which connects the fetus with the fetal surface of the placenta and it normally contains two arteries and one vein surrounded by Wharton’s jelly, all enclosed in a layer of amnion [1]. The umbilical cord appears twisted because of fetal movement or unequal growth of vessels. Developmentally, the umbilical cord is completely fetal in origin [2]. The cord deserves attention as early as the first trimester as the probability of identifying congenital anomalies will be much higher with careful cord assessment in the earlier period of gestation. The umbilical cord develops from the body stalk and has a different structure at different stages of development but the fully developed umbilical cord is about 45–50 cm in length and 1–2 cm in diameter [2].

Normally umbilical cord inserts at the center or near the center (eccentric) of the placental disc tissue [3]. But sometimes it can attach to the periphery of the placental tissue (marginal insertion) or, otherwise the umbilical cord and its vessels inserted between the chorioamniotic membrane of the placenta rather than to the tissue [4]. Variations in the site of the insertion of the umbilical cord are thought to result from the process known as trophotrophism in which the chorionic frondosum or the early placenta migrates with advancing gestation to ensure a better blood supply from a more richly vascularized area [1].

Abnormalities in the development and site of the insertion of the umbilical cord can cause problems that have the potential to affect maternal and fetal health and wellbeing [3]. Marginal cord insertion (MCI), with an incidence ranging from 2 to 25% [5], is thought to be associated with different risk factors like bleeding in pregnancy, advanced maternal age, pregnancies conceived with the aid of artificial reproductive technology, maternal chronic disease, and drug abuse during pregnancy [6].

Marginal cord insertion leads to intrauterine growth retardation (due to a decrease in blood flow to the developing fetus), low birth weight, low Apgar score [7], preterm labor, and development of velamentous type [6, 8]. It is also a known risk factor for the occurrence of congenital abnormalities, compression of fetal vessels, and stillbirths (if MCI present in the cervical outlet of the uterus), and excessive hemorrhage (if associated with vasa praevia) [6]. Directions about MCI and related issues are expected from World Health Organization in general and the Ethiopian Federal Ministry of health in particular to solve associated problems. Even though the burden of MCI is high to this end worldwide, there is a clear gap in measuring the magnitude, identifying the risks, and birth outcomes of MCI in the Ethiopian community. For this reason, this study aimed to measure the magnitude, state the possible risk factors, and identifying the adverse birth outcomes of MCI in the University of Gondar Comprehensive Specialized Hospital (UOGCSH), Northwest Ethiopia.

## Methods

### Study period and setting

The study was conducted from May 01, 2020, to July 30, 2020, at the UOGCSH, Maternity and Neonatal Ward. The UOGCSH is located in Amhara regional state, 738 km far from the capital city of Ethiopia, Addis Ababa, to the Northwest in Gondar town. University of Gondar Comprehensive Specialized Hospital has 611 patient’s beds, which acts as the referral center for more than twelve districts and general hospitals in the area. The hospital serves a population of more than seven million across the region. It has a range of specialties including Internal medicine, Pediatrics, Surgery, Gynecology, Radiology, Ophthalmology, Psychiatry, HIV care, and an outpatient clinic. Gynecology and Obstetrics is one of the major departments in the school of medicine. This department has 13 senior Gynecologist, 42 residents, and 127 midwives. An average of 845 mothers gives birth per month in this department. The Department of Gynecology and Obstetrics, in the UOGCSH, runs one labor and delivery ward, two postpartum maternity wards, one high-risk ward, one gynecology ward, one uro-gynecologic ward, one safe abortion and postpartum follow-up clinic (known as Michu-clinic), four gynecologic outpatient departments, one neonatal ward, and six antenatal care clinics (ANC) including PMTCT and feto-maternal consultation room.

### Study design and population

The institution-based cross-sectional study design was conducted. All singleton births at the UOGCSH in the Maternity and Neonatal Ward were the source population. All singleton births which met the inclusion criteria within the study period at the UOGCSH Maternity and Neonatal Ward were the study population.

### Eligibility criteria

All singleton births were included in the study.

Placenta specimens without intact umbilical cord and with externally identifiable pathology, bifurcated umbilical cord before its insertion, seriously ill mothers (if unable to communicate), and velamentous type of placental cord insertion were excluded from the study.

### Variables of the study

#### Dependent variable

Marginal cord insertion and adverse birth outcomes are the dependent variables.

#### Independent variables

Sociodemographic characteristics are advanced maternal age, residence, income, educational level, and marital status. Obstetrical and pregnancy-related variables include previous cesarean delivery, parity, prior termination, fertility problems, previous miscarriage, bleeding during pregnancy, placenta praevia, sex of the fetus, current preeclampsia, and gestational diabetes mellitus (GDM). Drugs and medicine variables include the use of the intrauterine contraceptive device (IUCD), smoking cigarettes, folic acid supplementation, and the consumption of alcohol. Maternal health-related factors are maternal diabetes, epilepsy, chronic hypertension, rheumatoid arthritis, and asthma.

### Sample size determination

The sample size was determined by using the single population proportion formula. The following assumptions were considered into account: Population proportion (*P* = 0.5), the margin of error (d = 0.05), and 95% confidence interval. It was calculated as n = (Zα/2)^2^ p (1-p)/d^2^, n = (1.96) (1.96) (0.5) (0.5)/ (0.05)^2^ = 384. By adding the 10% non-response rate, the final sample size calculated was 422.

### Sampling technique

A systematic random sampling technique was conducted to select study participants. By considering the expected total number of delivery services on two consecutive months by average (1690), we determined the sampling interval of participants = 4 (K=N/*n* = 1690/422). Thus, every 4th mother and her placenta with the attached umbilical cord were enrolled in the study by starting at the randomly selected (4th) newly delivered eligible mother. The participants were selected 24 h a day at the time of delivery.

### Operational definitions

#### Marginal cord insertion

It is diagnosed when the distance from the umbilical cord insertion site to the nearest edge of placenta is ≤2 cm.

#### Adverse birth outcomes

It is the occurrence of one of the following events; low birth weight, preterm birth, abruptio placenta, transfer to neonatal intensive care unit (NICU), low Apgar score, stillbirth, malformation, and emergency cesarean delivery (ECD).

### Data collection tools and procedure

The data were collected through the diagnosis of MCI, face-to-face interview (with a structured questionnaire), and chart review using a checklist (performed to get recorded data). The mothers’ condition, pregnancy, and birth outcome-related data were gathered using a structured questionnaire, which was adapted from different literature and modified according to our study objectives. Notably, the validity of the questionnaire has been ensured through a pretest study and expert discussion with different professional experts. Consequently, modifications have been carried out based on expert suggestions and the outputs of the pretest study. Besides, the questionnaire was assessed for reliability, clarity, content, and flow of the questionnaire following the collection of data in the pre-test study. The data from the pretest study was not included in the final analysis. The data collection questionnaire was first prepared in English and translated to Amharic and then back to English.

The data were collected by trained M.Sc. Midwives from the UOGCSH under the supervision of a Gynecology resident from the same institution.

After the placenta is delivered, the mother, neonate, and placenta were transferred into isolated room in the postpartum maternity ward. Then, the course and insertion site of the umbilical cord was noticed. The distance from the placental edge to the umbilical cord insertion site was also measured by using a tap meter. Following this, the diagnosis of MCI was made if the distance from the cord insertion site to the nearest edge of the placenta is ≤2 cm. The participants were recruited 24 h a day and the data were collected both day and night in the postpartum maternity ward.

### Data quality control and management

Before the data collection, to assure the quality of data, the data collection questionnaire was checked for clarity, understandability, uniformity, and completeness. The training was given to data collectors for two days about the objectives, process of data collection, and standard operating procedure. The pretest was carried out in 5% of the sample size before the actual data collection time in Koladiba primary hospital. Necessary adjustments and important amendments were maintained based on the pretest result. Everyday close supervision was undertaken by the trained supervisor and every other day by the principal investigator. Necessary feedbacks were offered to data collectors the next morning before data collection and the quality of equipment was also checked to ensure accuracy.

### Data processing and analysis

The collected data were checked manually for its completeness, coded, and entered into Epi Data version 3.1 statistical packages then exported to SPSS Version 20 for further cleansing and analysis. Inconsistent values were double-checked against the filled data extract format and corrected as necessary. Frequency and percentage tables were used to represent the results of categorical variables and means and standard deviations to represent continuous variables. Bi-variable and multivariable logistic regression analyses were used to determine the association of independent variables with the dependent variables. Variables with *P*-value< 0.2 in the bi-variable analysis were entered into a multivariable logistic regression model to identify the important determinants by controlling confounders. Odds ratios with 95% confidence interval were computed to identify the strength of associations, and statistical significance was declared if *P*-value < 0.05. The model was checked using the Hosmer–Lemeshow goodness of fit test.

### Ethical considerations

Ethical clearance was obtained from the ethical review committee of the School of Medicine, College of Medicine and Health Sciences, the University of Gondar (Ref. No 1879/ 02/ 2020). An official letter was submitted to UOGCSH, Gynecology, and Obstetrics Department. Finally, after having permission from the Maternity and Neonatal Ward coordinator; the mothers, from whom the specimens were obtained, were informed about the purpose and benefit of the study along with its procedure and their right to refuse. Furthermore, the study participants were reassured for the attainment of confidentiality (and privacy), and informed written consent was obtained from each mother at the time of data collection. Health education was provided to each participant following the data collection and mothers with adverse birth outcomes were well reassured and helped to have an appropriate management plan. We can confirm that all methods were performed in accordance with the relevant guidelines and regulations.

## Result

### Socio-demographic characteristics of the study participants

A total of 421 mothers participated in the study with a response rate of 99.76%. The age of respondent mothers in this study ranged between 16 and 45 years with a mean age of 27 years and SD 6.19. Most of the mothers, 87.4%, came from urban. Eighty-nine percent of the respondents were married, and orthodox Christian is the predominant religion in the study participants which accounts for 83%. About 10.9% of the respondents were illiterate whereas 49.9% were certified with a diploma or above. Eighty percent of the respondent mothers were either government employees or housewives (Table 1).

**Table 1:** Descriptive statistics of socio-demographic characteristics of mothers in the University of Gondar Comprehensive Specialized Hospital, Northwest Ethiopia, 2020 (*n* = 421)

Variables	Categories	Frequencies	Percent
Maternal age	< 35	366	87.0
	≥ 35 years old	55	13.0
Residence	Urban	368	87.4
	Rural	53	12.6
Marital status	Married	375	89.0
	Unmarried	37	8.9
	Divorced	9	2.1
Religion	Orthodox	349	83.0
	Muslim	69	16.3
	Protestant	3	.7
Educational Status	Illiterate	46	10.9
	Read and write	32	7.6
	Complete primary education	48	11.4
	Complete second education	85	20.2
	Diploma and above	210	49.9
Maternal job	Housewife	165	39.2
	Student	29	6.9
	Government employee	171	40.6
	Private business	56	13.3
Income (in Ethiopian birr)	≤ 1210	79	18.8
	1211–8970	233	55.3
	> 8970	109	25.9
Ethnicity	Amhara	376	89.3
	Qimant	19	4.5
	Tigre	10	2.4
	Others	16	3.8
Family size	< 4	351	83.4
	4–7	67	15.9
	> 7	3	.7

### Pregnancy and obstetric related characteristics

As described in Table 2, 74.3% of the study participants were multiparous. And from the multiparous women, 9.9% had at least a one-time cesarean delivery history. Nearly 13% of the respondents had a history of either miscarriage or stillbirth. Infertility had been a challenge for 7.6% of mothers before the last pregnancy. About 7.8 and 4.0% of participants in this study had vaginal bleeding and placenta praevia, respectively in the current pregnancy. About half of the mothers in this study had a fetus of the female sex, and 0.5% of the umbilical cords were found with a single artery (Table 2).

**Table 2:** Descriptive statistics of pregnancy and obstetrical characteristics of mothers in the University of Gondar Comprehensive Specialized Hospital, Northwest Ethiopia, 2020 (*n* = 421)

Variables	Categories	Frequencies	Percentages
Parity	Primiparous	108	25.7
	Multiparous	313	74.3
Previous CS	No	390	92.6
	Yes	31	7.4
Stillbirth	No	393	93.4
	Yes	28	6.6
Miscarriage	No	394	93.6
	Yes	27	6.4
Fertility problem	No	389	92.4
	Yes	32	7.6
Placenta praevia	No	404	96.0
	Yes	17	4.0
Vaginal bleeding	No	388	92.2
	Yes	33	7.8
Sex of fetus	Female	200	47.5
	Male	221	52.5
Number of umbilical arteries	One	2	.5
	Two	419	99.5
Current preeclampsia	No	388	92.2
	Yes	33	7.8
GDM	No	393	93.3
	Yes	28	6.7

*GDM* Gestational diabetes mellitus

### Maternal medicine and drug usage

As presented in Table 3 below, 16.2% of mothers had a history of alcohol consumption but no mother was found with a history of smoking during and before the pregnancy. Of the entire respondent mothers, 94.3% took folic acid supplementation during the pregnancy time, and 8.1% of the women had a history of IUCD usage (Table 3).

**Table 3:** Descriptive statistics of medicine and drug characteristics of mothers in the University of Gondar Comprehensive Specialized Hospital, Northwest Ethiopia, 2020 (*n* = 421)

Variables	Category	Frequencies	Percent
Alcohol consumption during pregnancy	No	353	83.8
	Yes	68	16.2
Smoking	No	421	100
	Yes	0	0
Folic acid supplementation	No	24	5.7
	Yes	397	94.3
Use of IUCD before pregnancy	No	382	91.9
	Yes	34	8.1

*IUCD* intrauterine contraceptive device

### Maternal medical conditions

Of all participants, 10.2, 2.9, 2.6, and 0.5% of participants were found hypertensive, asthmatic, diabetic, and epileptic, respectively (Table 4).

**Table 4:** Descriptive statistics of maternal chronic disease characteristics in the University of Gondar Comprehensive Specialized Hospital, Northwest Ethiopia, 2020 (*n* = 421)

Variables	Category	Frequencies	Percent
Chronic hypertension	No	378	89.8
	Yes	43	10.2
Asthma	No	409	96.1
	Yes	12	2.9
Maternal diabetes	No	410	96.4
	Yes	11	2.6
Epilepsy	No	419	99.5
	Yes	2	0.5
Rheumatoid arthritis	No	421	100
	Yes	0	0

### The magnitude of marginal cord insertion

The magnitude of MCI was 6.4% with a 95% CI of 4.4–8.8%. The rest 93.6% of the placentas had normal cord insertion with 53.3% were eccentric and 40.3% were central.

### Adverse birth outcomes

Low birth weight (LBW), accounts for about 13% of the study population, and 9.3% of births were preterm. The prevalence of ECD during the study period was 4.3%. About 6% of the newborn were transferred to NICU and 4% was born died. The prevalence of abruptio placenta in this study was 2.4% (Table 5).

**Table 5:** Descriptive statistics of adverse birth outcomes in the University of Gondar Comprehensive Specialized Hospital, Northwest Ethiopia, 2020 (*n* = 421)

Variables	Category	Frequencies	Percent
Birth weight	≥ 2500 g	365	86.7
	< 2500 g	56	13.3
GA at birth	≥ 37 weeks	382	90.7
	< 37 weeks	39	9.3
Abruptio placenta	No	411	97.6
	Yes	10	2.4
Transfer to NICU	No	397	94.3
	Yes	24	5.7
Apgar score at 1 min	≥ 7	400	95
	< 7	21	5
Apgar score at 5 min	≥ 7	395	93.9
	< 7	26	6.1
Stillbirth	Yes	404	96
	No	17	4
Malformation	No	419	99.5
	Yes	2	0.5
ECD	No	403	95.7
	Yes	18	4.3

*GA* gestational age, *NICU* neonatal intensive care unit, *ECD* emergency cesarean delivery

### Risk factors associated with marginal cord insertion

In the binary logistic regression analysis, advanced maternal age, primiparity, previous cesarean section, use of IUCD before pregnancy, maternal chronic hypertension, placenta praevia, and vaginal bleeding during pregnancy were identified variables as the candidate for multivariable analysis at *P*-value less than 0.2 for MCI (Table 6).

**Table 6:** Result of bivariate and multivariable logistic regression for risks associated with MCI in the University of Gondar Comprehensive Specialized Hospital, Northwest Ethiopia, 2020

Variables	Category	Marginal cord insertion		COR (95% CI)	AOR (95% CI)
		No	Yes		
Maternal age	< 35	351	15	1	1
	≥35	43	12	4.76 (2.7–11.58)***	2.24 (1.35–11.08)***
Parity	Multiparous	300	13	1	1
	Primiparous	94	14	2.54 (1.88–7.35) * ^*^	1.98 (1.37–8.69)**
Previous CD	No	369	21	1	1
	Yes	25	6	4.97 (2.14–12.06)***	2.51 (1.43–10.21)*
IUCD	No	367	20	1	1
	Yes	27	7	5.68 (2.32–12.27)***	2.22 (1.36–12.30)**
Chronic hypertension	No	359	19	1	1
	Yes	35	8	3.46 (2.03–9.83)***	3.07 (1.66–9.76)**
Vaginal bleeding	No	364	24	1	1
	Yes	20	3	2.11 (0.95–6.42)	1.01 (0.53–6.55)
Placenta praevia	No	380	24	1	1
	Yes	14	3	6.29 (2.20–18.27)*	2.01 (0.14–12.39)

*MCI* marginal cord insertion, *CD* cesarean delivery, *IUCD* intrauterine contraceptive device, *AOR* Adjusted Odd Ratio, *CI* Confidence Interval, *COR* Crude Odd Ratio; ***p-value = < 0.001, **p value = < 0.01,*p-value = < 0.05

Hosmer and Lemeshow test goodness of fit = 0.414

In multivariable logistic regression analysis, advanced maternal age, previous cesarean section, use of IUCD before pregnancy, maternal chronic hypertension, and primiparity were variables that showed statistically significant association with MCI.

Accordingly, primiparous mothers were nearly two times more likely to have MCI compared to their counterparts (AOR = 1.98 (95% CI = 1.37–8.69). The odds of having MCI in mothers with advanced age (> = 35) was 2.24 times higher (AOR = 2.24, 95% CI = 1.35–11.08) than those with lower age. A mother with a previous history of cesarean delivery (CD) showed a 2.5 fold increment in developing MCI (AOR = 2.51, 95% CI = 1.43–10.21) compared to a mother with no history of cesarean delivery. Women who have had chronic hypertension had 3.07 times (AOR = 3.07, 95% CI = 1.66–9.76) higher risk to develop MCI than their counterparts. Besides, mothers who had been using IUCD before pregnancy (AOR = 2.22, 95% CI =1.36–12.30) were around two times more likely to have MCI compared to mothers who did not use IUCD (Table 6).

### Adverse birth outcomes associated with marginal cord insertion

The data presented in Table 7 indicate that MCI was associated with an increased risk of low birth weight (OR = 4.73, 95% CI = 2.27–9.85), preterm birth (OR = 4.36, 95% CI = 1.92–9.85), and ECD (OR = 5.79, 95% CI = 2.04–16.47) compared to normal cord insertion. We also compared low birth weight in term (*n* = 392) newborns and identified the rate of low birth weight is still high in MCI relative to normal cord insertions (OR = 3.21, 95% CI = 1.34–5.97).

**Table 7:** Result of bivariate logistic regression for MCI associated with adverse birth outcomes in the University of Gondar Comprehensive Specialized Hospital, Northwest Ethiopia, 2020

Cord insertion site	Conditions	No	Yes	OR	95% CI
Normal	Low birth weight	349	45		
Marginal		16	11	4.73	2.27–9.85***
Normal	Preterm birth	362	32		
Marginal		20	7	4.36	1.92–9.85**
Normal	ECD	379	15		
Marginal		24	3	5.79	2.04–16.47***
Normal	Abruptio placenta	381	13		
Marginal		26	1	2.89	.77–10.88
Normal	Transfer to NICU	372	22		
Marginal		26	1	2.25	.725–7.00
Normal	Apgar score at 1 min < 7	375	19		
Marginal		25	2	1.73	.48–6.19
Normal	Apgar score at 5 min < 7	360	23		
Marginal		25	2	1.34	.38–4.69
Normal	Stillbirth	368	15		
Marginal		26	1	2.25	.62–8.24

*OR* Odd Ratio, *CI* Confidence Interval, *** *p*-value = < 0.001, **p value = < 0.01, *NICU* neonatal intensive care unit, *ECD* emergency cesarean delivery

After adjusting for maternal age, parity, hypertension, current preeclampsia, and GDM in a multivariable logistic regression, MCI remained associated with low birth weight (AOR = 2.89, 95% CI = 1.23–6.80), preterm birth (AOR = 4.00, CI = 1.44–11.14) and ECD (AOR = 3.68, 95% CI = 1.03–13.81) (Table 8).

**Table 8:** Result of multivariable logistic regression for MCI and maternal characteristics associated with adverse birth outcomes in the University of Gondar Comprehensive Specialized Hospital, Northwest Ethiopia, 2020

Predictor variables		Outcome: Low birth weight		Outcome: Preterm birth		Outcome: ECD
		OR (95% CI)	*P*-value	OR (95% CI)	*P*-value	OR (95% CI)
Maternal age	< 35	1.00	.35	1.00	.84	1.00
	> = 35	1.47 (0.65–3.32)		.90(.31–2.61)		1.43(.40–5.05)
Parity	Primiparous	1.00	.067	1.00	.50	1.00
	Multiparous	1.81 (0.95–3.45)		.74(.31–1.75)		0.86 (0.27–2.69)
PCI	Normal	1.00	.01	1.00	.008	1.00
	Marginal	2.89 (1.23–6.80)		4.00 (1.44–11.14)		3.68 (1.03–13.81)
Hypertension	No	1.00	.23	1.00	.60	1.00
	Yes	1.67(.71–3.92)		1.34(.44–4.07)		2.62 (0.78–8.77)
Preeclampsia	No	1.00	.007	1.00	<.001	1.00
	Yes	9.29 (1.8646.34)		8.20 (3.34–17.54)		3.80 (0.50–28.71)
GDM	No	1.00	.13	1.00	.08	1.00
	Yes	.24(.04–1.52)		1.45(.98–2.95)		0.72 (0.87–5.97)

*PCI* placenta cord insertion, *GDM* gestational diabetes mellitus, *CI* confidence interval, *OR* odds ratio, *ECD* emergency cesarean delivery

## Discussion

The present study, aimed to assess the magnitude, risk factors, and adverse birth outcomes of MCI, presents the report of 421 singleton births in the UOGCSH, Northwest Ethiopia. The magnitude of MCI in this study was 6.4%. Advanced maternal age, primiparity, maternal chronic hypertension, previous cesarean delivery, and use of the intrauterine contraceptive device before pregnancy are risk factors for MCI. Pregnancies complicated by MCI are at higher risk to develop low birth weight, preterm birth, and emergency cesarean delivery than normal cord insertion.

A population-based study conducted among all singleton births at gestational weeks 16–45 in Norway during the period 1999–2009 revealed that the prevalence of MCI was 6.3% [9]. Similarly, in the present study, the prevalence of MCI was 6.4%. The prevalence of MCI in this study was also in line with the studies done in Ireland, France, and India [5, 10, 11]. Nonetheless, the prevalence of MCI in the present and Norwegian study was higher than the previous reports from India and Pakistan [12, 13]. The possible explanation for the higher prevalence in the present study relative to the Indian study might be due to a difference in the selection of study participants. The Indian study did not include mothers with a chronic illness which might increase the prevalence of MCI [9]. On the other way, the Indian study excluded fetal anomalies from the study which might result from MCI [5]. The higher prevalence in the current study compared to the Pakistan report might be due to the increasing incidence of risks that have been associated with MCI. Such as maternal chronic disease (chronic hypertension), cesarean or manual vacuum aspiration deliveries, and IUCD practice [8]. However, the prevalence of MCI in the present study was lower than the studies done in India and the USA [14, 15]. The difference in the prevalence value of MCI between the present and USA study is probably due to methodological difference since the American study used sonographic evaluation to measure the outcome, there might be the occurrence of a false-negative sonogram, placental remodeling, and technical error which might lead to a high prevalence of MCI [14]. Besides, it might also be due to the absence of multiple gestations and assisted reproductive technology user respondents in our study population [9]. The higher MCI prevalence in the Indian study relative to the current study might be due to outcome measurement variation since the Indian study did not use specific outcome measurement criteria while we only consider cord insertion ≤2 cm to the nearest of the placental edge as MCI.

In the present study, we have found a statistically significant strong positive association between maternal age and MCI. In such a way that mothers with advanced age possibly faced a risk of MCI by two folds (Table 6). Interestingly, our finding is strongly supported by the study conducted elsewhere [9]. The reason why MCI is common in advanced age group mothers (> = 35) is probably due to uterine hypoxia. Since uterine hypoxia is a common scenario in advanced age group mothers [16], it induces Trophotrophism (the migration of early placental cells from one part of the uterus to other parts to search for nutrition), which leads to MCI.

Like other studies done in Norway, our study revealed that primiparity was a significant predictor of MCI. Therefore, the first pregnancy was at a higher risk of developing MCI. Since different studies revealed a direct association between placenta praevia and MCI [8, 17], the possible explanation for the higher risk of primiparity compared to multiparity for MCI is due to the higher incidence of placenta praevia in primiparity [18]. In our study, the prevalence of placenta praevia was 7.4 and 2.8% among primiparous and multiparous, respectively.

In this study, mothers with chronic hypertension had 3.07 times higher odds of developing MCI compared to their counterparts. This result is consistent with previous studies from Norway and Israel [9, 18]. Jain A. and collaborators stated a strong association between anomalous cord insertion and altered placental shape [19]. Since altered placental shape, as a result of repeated branching of uteroplacental vascular trees, occurred due to chronic hypertension [20], our report of a positive association between chronic hypertension and MCI is indirectly supported.

The present study indicated that previous cesarean delivery had about two and half folds of developing MCI compared with mothers who did not have a history of cesarean delivery (Table 6). A study in the Norwegian population also showed an association between previous experience of cesarean delivery and anomalous cord insertion [9]. This is most likely to be related to abnormal placentation, which is associated with uterine scarring from prior cesarean section [21].

In our study, we have found that the usage of IUCD before conception will put the mother two times higher risk of developing MCI compared to their counterparts. Some studies observed a gradual endometrial thinning after the use of IUCD [22]. Conception on a thin endometrium might lead to asymmetrical placental development from the point of cord insertion, the placenta being less developed where the endometrium is not well regenerated [23].

One of the causes of anomalous cord insertion is abnormal vessel formation or abnormal placement of early placental cell during implantation [24], this indirectly affects placenta and fetal development and growth which lead to adverse birth outcomes [25]. Thus, the present study confirms the previous finding of increased risk of preterm birth in pregnancies with MCI [13]. Our finding of a positive association between MCI and low birth weight is supported by a study done in the USA [26]. Sophie Brouillet et al. declared MCI causes low birth weight because from a geometrical and mathematical perspective MCI could not ensure better and adequate blood supply to the fetus [11] which in turn leads to underweight.

Similar to the findings in other settings [9], we observed that ECD is 3.68 times in births with MCI compared to normal cord insertion. This is may be due to fetal distress (the second most cause of ECD [27]) as a result of inadequate blood supply.

### Strength and limitations of the study

#### Strength of the study

The study is the first of its kind in the study area and Ethiopia as well. The direct measurement of the umbilical cord insertion site was performed precisely to the nearest decimal and accordingly recorded by well-trained data collectors under the close supervision of the investigators.

#### Limitations of the study

This study did not consider some potential risk factors like preconception body mass index and cord insertion type of previous pregnancy due to the absence of recorded data as the habit of preconception medical consultation is uncommon in Ethiopia. Since this study was conducted in health institutions, the result of the study might not be generalized to the entire population. Cross-sectional nature of the study (since MCI is a rare case), and comparison of results with developed countries (western countries) are among the limitation of this study.

## Conclusions

The prevalence, most of the risk factors, and adverse birth outcomes in the present hospital-based cross-sectional study are in accordance with previous studies. Primiparous women, advanced aged women, women with chronic hypertension, women who have been using IUCD before pregnancy, and a mother with a history of previous cesarean delivery are at high risk to develop MCI. Low birth weight, preterm birth, and emergency cesarean delivery are adverse birth outcomes directly associated with MCI in this study.

## Recommendations

- Prenatal detection of MCI should be performed routinely especially for those pregnancies at high risk of cord insertion anomaly during antenatal care follow-up.
- Data about pregnancy and obstetrical history including the cord insertion type from pre-pregnancy to delivery should be recorded appropriately.
- Knowledge, attitude, and practice of health care workers towards MCI should be assessed.
- Researchers should conduct a large-scale study (multi-center, prospective study) using the current study as baseline data.

## Data Availability

The data sets used and/or analyzed during the current study are available from the corresponding author on reasonable request.

## Abbreviations

AOR: Adjusted odds ratio
CD: Cesarean delivery
ECD: Emergency cesarean delivery
GDM: Gestational diabetes mellitus
IUCD: Intrauterine contraceptive device
LBW: Low birth weight
MCI: Marginal cord insertion
NICU: Neonatal intensive care unit
UOGCSH: University of Gondar comprehensive specialized hospital
